# Behavioral and Psychological Predictors of Multiple-Perpetrator Rape Proclivity: A Community Sample Study of Men

**DOI:** 10.5964/sotrap.13697

**Published:** 2025-06-12

**Authors:** Saad Iqbal, Alexandra M. Zidenberg, Michelle Schwier

**Affiliations:** 1 School of Graduate Psychology, Pacific University, Hillsboro, OR, USA; 2 School of Criminology, University of Montreal, Montreal, Quebec, Canada; 3 Department of Psychology, Western University, London, Ontario, Canada; Centre for Criminology (Kriminologische Zentralstelle – KrimZ), Wiesbaden, Germany

**Keywords:** multiple-perpetrator sexual rape, rape proclivity, male offenders, M-PRIS

## Abstract

Most of the sexual violence research focuses on incidents involving a single offender, yet one-fourth to one-third of rapes involved multiple offenders (Horvath & Kelly, 2009). The present study aimed to build upon the multiple-perpetrator rape (MPR) literature by investigating potential correlates associated with a proclivity for MPR and reconfirming prior findings. Community men completed a series of questionnaires that included the Multiple-Perpetrator Rape Interest Scale, the UCLA Loneliness Scale: Short-Form, the Buss–Perry Aggression Questionnaire: Short Form, the Sexual Fantasy Questionnaire, the Anger Rumination Scale, the Measure for Assessing Subtle Rape Myths, and the Self-Report Psychopathy-III: Short Form. The strongest relationship for M-PRIS was SFQ Sado-Masochistic (*r* = .79, *p* < .001). In a multiple linear regression, results showed a significant model, *F*(6, 108) = 28.6, *p* < .05, which explained 61.4% of the variance in a proclivity for MPR. Specifically, BPAQ Total, SFQ Total, and ARS Total were significant (*sr*^2^ = .0543, 0.0499, 0.0488, respectively). One implication is for clinicians to target various types of aggression, deviant sexual fantasies, and anger rumination in therapy among those with an interest to commit MPR to potentially reduce the urge to commit the action. Educational and preventive initiatives aimed at addressing sexual violence behaviors may also gain insights into individuals prone to engaging in such behaviors, where these programs seek to diminish the likelihood of MPR occurrences.

## Highlights

- The current study provides information on correlates of various psychological characteristics and attitudes with a proclivity to multiple-perpetrator rape.
- Specifically, multiple linear regressions indicated aggressive behavior tendencies, deviant sexual fantasies, and anger rumination significantly correlated with a proclivity to MPR.
- Psychopathy, loneliness, and rape-supportive cognitions were found not to be significant in the multiple linear regression model.

The characteristics and attitudes of individuals who may potentially commit multiple-perpetrator rape (MPR)–defined as any sexual assault involving two or more offenders–is a woefully under-researched area (Alleyne et al., 2014; Horvath & Kelly, 2009). Most sexual violence research has grouped MPR offenders with lone sexual offenders, who may potentially have unique characteristics and attitudes as compared to lone offenders (da Silva et al., 2015; Harkins & Dixon, 2010). The distinction between the two groups is important to reduce the prevalence and recidivism of lone and multiple-perpetrator sexual violence. Fortunately, there is a small body of literature that has investigated the differences in demographics and offense-related information among lone and MPR offenders.

In a systematic review and meta-analysis of the characteristics of MPR offenders, Bamford and colleagues (2016) found several differences between lone and multiple offenders who commit rape. Those involved in multiple-perpetrator offenses were fairly young with most being in their early 20s, of Black/African Caribbean ethnicity, and the most common grouping was a pair. Compared to their lone perpetrator counterparts, those involved in multiple-perpetrator offenses had lower rates of prior general and sexual convictions. Multiple-perpetrator sexual offenders were more likely to offend against strangers and outdoors while lone offenders were more likely to know their victims and approached them indoors. Violence and weapon usage were more prevalent in multiple-perpetrator sexual offenses as well. With regards to victim characteristics, multiple-perpetrator sexual offenders' victims tended to be White, younger, resist more, and have lower socioeconomic status compared to lone sexual offenders' victims. Of note, lone offenders were found to be more likely than multiple-perpetrator offenders to recidivate (Bamford et al., 2016). The varying differences from past studies demonstrate the need to further research in understanding the clear, unique differences between lone and multiple-perpetrator offenders.

Furthermore, Lim (2018) investigated the relationship between the number of perpetrators in a sexual assault, the perceived level of victim blame, and victim resistance through vignettes. Lim (2018) found that perceived victim blame did not positively correlate with the number of perpetrators involved in a sexual assault. Lone offender groups were attributed more blame than multiple-offender groups, which is not in line with previous research (Adolfsson et al., 2020). Furthermore, as the level of victim resistance diminished, there was an increase in the perceived victim blaming, revealing an inverse relationship (Lim, 2018). Understanding varying contexts and attributions of MPR is important in understanding the phenomenon holistically and its impact on society and individuals.

## Explaining and Measuring Interest in MPR

The most widely accepted theory to explain MPR is the Multi-Factorial Theory of Multiple-Perpetrator Sexual Offending, which proposes that MPR occurs due to an interaction of individual, sociocultural, and situational factors (Harkins & Dixon, 2010, 2013). Individual factors consist of personality traits, developmental factors, and sexual preferences, with leadership and deviant sexual interests theorized as being the most impactful. Sociocultural factors consist of cultural norms, myths, values, and beliefs, with rape culture, rape myths, and patriarchy being the most important in increasing the likelihood of MPR. Lastly, situational factors consist of the setting in which MPR may occur, such as pedophile organizations or war. The current study focused on individual and sociocultural factors.

In order to further understand MPR, Alleyne and colleagues (2014) developed a novel, vignette-based, proclivity measure called the Multiple-Perpetrator Rape Interest Scale (M-PRIS), which assesses sexual arousal to MPR, behavioral propensity towards MPR, and enjoyment of MPR. The M-PRIS has demonstrated strong psychometric properties, specifically in its high internal reliability and strong test-retest reliability. Prior research with the M-PRIS has demonstrated that certain constructs hold positive associations with a proclivity for multiple-perpetrator rape, such as deviant sexual interests (i.e., high-risk sexual fantasies), rape-supportive cognitions, and antisocial attitudes (Alleyne et al., 2014; Palermo et al., 2019) while others do not, such as a dominant personality (Petrella & Harkins, 2021). These results, particularly related to deviant sexual interests and rape-supportive cognitions lend support to the Multi-Factorial Theory of Multiple-Perpetrator Sexual Offending (Harkins & Dixon, 2010, 2013), which posits that deviant sexual interests and rape-supportive cognitions are key factors underpinning MPR offences. Thus, subscales of deviant sexual interests and rape-supportive cognitions should be further investigated in their relationship with MPR.

## Individual Factors of MPR

Few studies have examined aggression levels within MPR. Woodhams and Cooke (2013) examined the relationship between suspect aggression and victim resistance in MPR. They found that victim resistance was significant and the type of rape (simultaneously versus sequential) was positively associated with suspect aggression. Additionally, larger group size was significantly associated with less victim resistance. Moreover, Hauffe and Porter (2009) compared lone and group sexual assault characteristics and found that group sexual assaults were more likely than lone sexual assaults to be hostile and violent in nature (i.e., use of violence to restrain the victim and hold the victim down, use of multiple weapons), involve multiple rapes by the same offender, and vaginal penetration. Consistent with prior research on group size and MPR, the findings suggest that MPRs consist of a higher degree of aggression (Hauffe & Porter, 2009; Woodhams & Cooke, 2013; Woodhams et al., 2020). Thus, aggression should be further investigated in their relationship with MPR.

Based on prior literature on sexual violence among lone offenders, we decided to investigate additional potential correlates that may potentially be related to MPR. The correlates map onto dynamic risk factors, which are factors that are potentially changeable (Ward & Fortune, 2016). First, loneliness and lack of intimacy have been extensively researched as potential dynamic risk factors clinically relevant in sexual offenders (Seidman et al., 1994; Wielinga et al., 2021). Marshall (2010) proposed that an interaction between poor childhood attachment style, lack of intimacy, and loneliness (both social and emotional) leads to vulnerability, which eventually leads one to sexually offend. MPR is inherently a group and social action (Harkins & Dixon, 2010), thus, perpetrators may feel a sense of camaraderie and belonging during and after committing the sexual offense. Moreover, the immense longing for social connections may lead individuals to sexually offend in a group versus alone, so they may form connections they did not have in childhood. Therefore, loneliness was investigated to further understand how it relates to MPR proclivity.

Another potential correlate of interest is anger rumination, particularly given that no research known to the authors has examined anger rumination and MPR specifically. Anger rumination consists of focusing attention on angry moods, recalling past anger experiences, and thinking about the causes and consequences of anger episodes (Sukhodolsky et al., 2001). In the sexual offense literature, anger rumination plays a key role in grievance thinking, which is a type of cognitive distortion wherein an individual feeling wronged and experiences a combination of hostility, rumination, and vengefulness (Barnett, 2011). Grievance thinking has been identified as a motivational factor for sexual murders (Wakeling & Barnett, 2011). Deficits in controlling one’s grievance thinking may lead to sexual offending as a way to obtain revenge on women who the individuals feel have wronged them. Thus, anger rumination was chosen to better understand how negative ruminative thoughts may lead to MPR. Through further understanding of how anger rumination plays a role in sexual offending, particularly MPR, theoretical and clinical implications may be drawn to further the grievance thinking model.

Lastly, psychopathy was of interest due to its mixed relationship with sexual offending, and the possibility of having a different relationship with MPR than lone sexual offenses (Darjee, 2019; Higgs et al., 2019; Moretti et al., 2024; Mouilso & Calhoun, 2013). Drawing from Hare’s (2003) four factor model (i.e., Interpersonal Manipulation, Callous Affect, Erratic Lifestyle, and Antisocial Behavior), MPR could theoretically be linked to psychopathy and its factors in multiple ways. Examples of Interpersonal Manipulation traits include pathological lying, consistently glib, manipulative, and untrustful. MPR offenders may lie and manipulate victims as a group to coerce victims into situations, where they cannot obtain help. Callous Affect traits include a lack of remorse and empathy, low affect, and tough-mindedness. MPR offenders tend to be more violent in nature when compared to other sexual violence, so having a lack of remorse and empathy would allow MPR offenders to commit the rape without internal conflicts. Erratic Lifestyle traits include impulsivity, recklessness, and thrill-seeking behaviors. Because MPR offers a sense of security in numbers, offenders may act more impulsively and recklessly, which may lead them to engage in MPR. Lastly, Antisocial Behaviors traits consist of criminal behaviors, aggressive acts, and hostility. Through the four-facet model of psychopathy, we hope to further understand how MPR may relate to psychopathic traits.

## Sociocultural Factors of MPR

Sociocultural factors of MPR are factors ingrained in our culture or community, such as rape-myth cognitions or negative stereotypical attitudes and beliefs about women. Examples of rape-myth cognitions include victim blaming, denial of harm, misunderstanding consent, and rationalizing the perpetrator's actions (Ryan, 2019). They perpetuate sexual violence by reducing the severity of rape and its impact on victims. In this study, we focus on rape-myth cognitions, as prior research shows a positive association between rape-myth cognitions with multiple-perpetrator rape proclivity (Adolfsson et al., 2020; Alleyne et al., 2014; Palermo et al., 2019). Moreover, higher levels of rape-myth cognitions have demonstrated to predict sexual violence perpetration (Bohner et al., 1998; Chiroro et al., 2004; O’Connor, 2021; Süssenbach & Euteneuer, 2024). This underlies the importance to understand rape-myth cognitions in the context of MPR, as individuals may reinforce each other’s beliefs, which may normalize and enable sexual violence, furthering a culture where sexual violence is more likely to occur and be excused.

## Characteristics of Individuals Who Are More Likely to Support Rape

Research has found that men rather than women are more likely to believe rape myths, as well as individuals who did not know the victim (Crall & Goodfriend, 2016). The researchers also discovered no significant difference in rape myth acceptance based on sexual orientation or level of post-secondary education.

More specifically, Johnson and Beech (2017) observed that there haven’t been many studies to date (*n* = 8) that compared the general public with sexual offenders and their rape myth acceptance rates. However, in their meta-analysis, the authors discovered that sex offenders in contrast to non-offenders had lower sex role satisfaction, high trait anxiety, and high trait anger. Consequently, there was no difference in rape myth acceptance by rapists and sexual murderers. In a group of exclusively rapists, intimacy and loneliness deficits were correlated with rape myth acceptance and hostility towards women.

Work by Fisher et al. (1999) used the UCLA Emotional Loneliness Scale to evaluate child molesters and prison officers on their emotional loneliness to see if there were differences by sample. The authors discovered that loneliness was indeed higher in the child molesters. In the same vein, offenders (murder, trafficking, robbery or theft) were higher in psychopathy on the PCL-R than non-offending male youths (Castellana et al., 2014).

These results indicate that there is empirical support for the speculation that offending samples are inherently different on beliefs and personality traits (e.g., rape myth acceptance, trait anger, emotional loneliness, and psychopathy) than nonoffenders. Thus, the current study looked to expand the literature within a non-offending sample.

## Current Study

The current study aims to build upon the MPR research by investigating potential correlates associated with a proclivity for MPR and reconfirming prior findings in the literature. Specifically, the correlates that will be examined are loneliness, aggressive behavior tendencies, anger rumination, deviant sexual interests, rape-supportive cognitions, and psychopathy. Through an understanding of the various correlates, potential dynamic risk factors and clinical targets may be identified to reduce prevalence and recidivism.

*Research Question:*

Do certain correlates have a stronger relationship than others with the proclivity for multiple-perpetrator rape?

## Method

### Procedure

The current study was reviewed and approved by King’s University College Research Ethics Review Committee. As part of a larger study, data was collected for individuals of all genders in a single Qualtrics survey (a high-level summary of the results for both men and women was presented in the newsletter for the Criminal Justice Section of the Canadian Psychological Association, Crime Scene; see Iqbal et al., 2024). Given that the main measure in this study (i.e., the M-PRIS) has only been validated in men, the decision was made to analyse the men and women in the sample separately (see Schwier et al., 2025, for the results from the women). This decision was made as there are distinct research questions and purposes for the two subsamples—the subsample with the men involves a replication and extension of previous research in the area and the subsample with the women involves a preliminary validation of the M-PRIS in this population and an exploration of correlates that have been previously tested with men. Unfortunately, there were not adequate numbers of non-binary individuals to allow for their inclusion in either output. The entire study was completed online via Qualtrics After giving consent, participants completed a demographics questionnaire, the Multiple-Perpetrator Rape Interest Scale, the UCLA Loneliness Scale: Short Form, the Buss-Perry Aggression Questionnaire: Short Form, the Sexual Fantasy Questionnaire, the Anger Rumination Scale, the Updated Measure for Assessing Subtle Rape Myths, and the Self-Report Psychopathy-III: Short Form with no time limit. Measures were in the aforementioned order, and titles of the measures did not appear in the survey. After completing the measures, participants were debriefed, thanked for their time, and participants could enter themselves into a raffle for the opportunity to win one of two $50 CAD gift cards.

### Participants

Participants were recruited through advertisements on social media (e.g., Twitter, Facebook, Reddit, Mastodon, and message boards dedicated to research), which were posted by the researchers. Participants were men from Canada and the United States (*N* = 115, *M*_Age_ = 30.09, *SD*_Age_ = 7.55). They were primarily White/Caucasian (60%), and the rest consisted of African-American/Black (6.96%), East Asian (3.48%), South Asian (4.35%), South East Asian (4.35%), Middle Eastern (4.35%), Native/Aboriginal/Indigenous (4.35%), Hispanic/Latino (4.35%), West Indian (1.74%), multi-racial - 5 (4.35%), and Other (1.74%). We obtained 451 responses and removed responses (*N* = 336) that had a very low completion rate, responded as “Trans,” “Non-Binary,” or “Female,” and/or completed the survey in less than ten minutes (see Schwier et al., 2025, for an analysis of the data from women [*n* = 182]).

### Measures

#### Multiple-Perpetrator Rape Interest Scale

The Multiple-Perpetrator Rape Interest Scale (M-PRIS; Alleyne et al., 2014) is a scenario-based instrument to assess support and interest in multiple-perpetrator rape. All scenarios presented in the M-PRIS have a woman as the victim, but the gender composition of the group is not specified. The scale consists of three intimidation and initiation scenarios, and respondents answer three questions per scenario. The first question asked participants how sexually aroused they would have been in each scenario ranked from 1 (“Not at all sexually aroused”) to 7 (“Very strongly sexually aroused”). The second question asked how likely it would be that they would behave in the way depicted by the scenario ranked from 1 (“Would have definitely not done the same”) to 7 (“Would definitely have done the same”). The final question asks how much they would have enjoyed getting their way in the situation ranked from 1 (“Would not enjoy it at all”) to 7 (“Would greatly enjoy it”). The M-PRIS has demonstrated strong internal consistency (all α coefficients > .75) and test-retest reliability (all correlations > .77; Alleyne et al., 2014). Total scores range from 6 to 126, with higher scores indicating a higher MPR proclivity. In this sample, Cronbach's alpha was .96.

#### UCLA Loneliness Scale: Short Form

The UCLA Loneliness Scale: Short Form (UCLA: SF; Hays & DiMatteo, 1987) is an 8-item instrument to assess one’s feelings of loneliness. Participants respond on a 4-point Likert-type scale: 1 ("Never") to 4 ("Often"). A total score is derived from summing the responses, which can range from 8 to 32 (e.g., "I feel isolated from others"). The UCLA: SF has demonstrated good psychometric properties (Hays & DiMatteo, 1987). In this sample, Cronbach's alpha was .73.

#### The Buss-Perry Aggression Questionnaire: Short Form

The Buss-Perry Aggression Questionnaire: Short Form (BPAQ: SF, Bryant & Smith, 2001) is a self-report instrument to measure aggressive behavior tendencies and has four subscales: Physical Aggression (e.g., “Given enough provocation, I may hit another person”), Verbal Aggression (e.g., “I often find myself disagreeing with people”), Anger (e.g., “I have trouble controlling my temper”), and Hostility (e.g., “Other people always seem to get the breaks”). Twelve items are rated on a Likert-type scale from 1 (“extremely uncharacteristic of me”) to 5 (“extremely characteristic of me”), with higher scores indicating a greater tendency for aggressive behavior tendencies. The BPAQ: SF has demonstrated strong psychometric properties (α = .70–.83; Bryant & Smith, 2001). In this sample, Cronbach's alpha for the BPAQ:SF Total was .80, Physical Aggression was .65, Verbal Aggression was .58, Anger was, and Hostility was .58.

#### Sexual Fantasy Questionnaire

The Sexual Fantasy Questionnaire (SFQ; Wilson, 1988) is an instrument to measure one’s sexual preferences, desires, and experiences. Each of the four subscales consists of ten questions: Exploratory (e.g., “mate-swapping, sex with someone of different race”), Intimate (e.g., “kissing passionately, intercourse with a loved partner”), Impersonal (e.g., “sex with strangers, watching others”), and Sado-Masochistic (e.g., “tying someone up, being whipped or spanked”). Participants respond to a 5-point Likert-type scale: 1 (“Never”), 2 (“Seldom), 3 “(Occasionally”), 4 (“Sometimes”), 5 (“Often”), and 6 (“Regularly”). Possible scores range from 0 to 160, with a higher score indicating strong sexual preferences and desires. The SFQ holds strong psychometric properties (Exploratory subscale, α = 0.84; Intimate subscale, α = 0.92; Impersonal subscale, α = 0.77, Sado-Masochistic subscale, α = 0.81; Skovran et al., 2010). In this sample, Cronbach's alpha for the SFQ Total was .93, Exploratory was .77, Impersonal was .85, Intimate was .80, and Sado-Masochistic was .77.

#### Anger Rumination Scale

The Anger Rumination Scale (ARS) is a measure that assesses four dimensions of anger rumination: Angry Afterthoughts (e.g., “After an argument is over, I keep fighting with this person in my imagination”), Thoughts of Revenge (e.g., “I have daydreams and fantasies of a violent nature”), Angry Memories (e.g., “I feel angry about certain things in my life”), and Understanding of Causes (e.g., “I analyze events that make me angry”; Sukhodolsky et al., 2001). Participants are asked 19 items and respond to their frequency of behaviors, ranging from 1 (“Almost Never”) to 4 (“Almost Always”). Possible scores range from 19 to 76, with higher scores corresponding to greater levels of anger rumination. The ARS has demonstrated good validity and reliability for the total (α = 0.93; Sukhodolsky et al., 2001). In this sample, Cronbach's alpha for the ARS Total was .91, Anger Afterthoughts was .82, Thoughts of Revenge was .69, Angry Memories was .78, and Understanding of Causes was .66.

#### Updated Measure for Assessing Subtle Rape Myths

The Updated Measure for Assessing Subtle Rape Myth (SRMA; McMahon & Farmer, 2011) is a 22-item instrument that assesses one’s level of rape myths acceptance. The SRMA consists of four subscales: She Asked for It (e.g., "When girls go to parties wearing slutty clothes, they are asking for trouble."), He Didn’t Mean To (e.g., "Rape happens when a guy’s sex drive gets out of control"), She Lied (e.g., “Rape accusations are often used as a way of getting back at guys”), It Wasn’t Really Rape (e.g., "If a girl doesn’t physically resist sex - even if protesting verbally – it can’t be considered rape"). Responses are given on a 5-point Likert-type response scale ranging from 1 (“Strongly Disagree”) to 5 (“Strongly Agree”), with higher scores indicating greater rape myths acceptance. The SRMA has demonstrated strong psychometric properties (α = .87; McMahon & Farmer, 2011). In this sample, Cronbach's alpha for the SRMA Total was .79, She Asked for It was .67, He Didn't Mean to was .71, It Wasn't Really Rape was .29, and She Lied was .69.

#### Self-Report Psychopathy: Short Form

The Self-Report Psychopathy-III: Short Form (SRP-III: SF; Paulhus et al., 2016) is a 29-item instrument that assesses psychopathic traits. The SRP-III: SF consists of four facets: Interpersonal Manipulation (e.g., “I would get a kick out of ‘scamming’ someone”), Callous Affect (e.g., “I never feel guilty over hurting others”), Erratic Lifestyle (e.g., “I keep getting in trouble for the same things over and over”), and Antisocial Behaviors (e.g., “I have threatened people into giving me money, clothes, or makeup”). Responses are given on a 5-point Likert-type scale from 1 (“Disagree Strongly”) to 5 (“Agree Strongly”), with higher scores indicating greater psychopathic traits. The SRP-III: SF has demonstrated strong psychometric properties in community samples (α =  0.90, ωh =  0.93; Gordts et al., 2017). In this sample, Cronbach's alpha for the SRP Total was .90, Interpersonal Manipulation was .76, Callous Affect was .71, Erratic Lifestyle was .71, and Antisocial Behaviors was .76.

## Results

### Means and Standard Deviations

Means and standard deviations were derived for all scales and their subscales (see Table 1). The means for the M-PRIS Total, UCLA Loneliness Total, BPAQ Total, BPAQ Physical Aggression, BPAQ Verbal Aggression, BPAQ Anger, BPAQ Hostility, ARS Total, ARS Angry Afterthought, ARS Thoughts of Revenge, ARS Angry Memories, ARS Understanding of Causes, SRMA Total, SRMA She Asked For It, SRMA He Didn’t Mean To, SRMA Wasn’t Really Rape, SRMA She Lied, SRP Total, SRP Interpersonal Manipulation, SRP Callous Affect, SRP Erratic Lifestyle, SRP Antisocial Behavior were below the midpoint of the scale. The means for the SFQ Total, SFQ Exploratory, SFQ Intimate, SFQ Impersonal, and SFQ Sado-Masochistic were above the midpoint of the scale.

**Table 1:** Means, Standard Deviations, and Pearson’s r for Exploratory Variables Relationship With Proclivity for MPR

Variable	*r*
M-PRIS Total	–
UCLA Loneliness Total	.33***
BPAQ Total	.68***
BPAQ Physical Aggression	.60***
BPAQ Verbal Aggression	.50***
BPAQ Anger	.49***
BPAQ Hostility	.49***
SFQ Total	.74***
SFQ Exploratory	.73***
SFQ Intimate	.44***
SFQ Impersonal	.66***
SFQ Sado-Masochistic	.79***
ARS Total	.50***
ARS Angry Afterthoughts	.39***
ARS Thoughts of Revenge	.50***
ARS Angry Memories	.42***
ARS Understanding of Causes	.43***
SRMA Total	.65***
SRMA She Asked For It	.59***
SRMA He Didn’t Mean To	.54***
SRMA Wasn’t Really Rape	.49***
SRMA She Lied	.19*
SRP Total	.63***
SRP Interpersonal Manipulation	.56***
SRP Callous Affect	.56***
SRP Erratic Lifestyle	.56***
SRP Antisocial Behavior	.48***

*Note.* *N* = 115.

*p < .05. **p < .01. ***p < .001.

### Correlations

There were positive, significant associations between all exploratory variables and the outcome variable, M-PRIS Total, that ranged from weak to strong (see Table 1 for Pearson’s *r*). Exploratory variables are the total scores and subscales for each measure used: UCLA: SF, BPAQ: SF, SFQ, ARS, SRMA, and SRP-III: SF. The strongest relationship for M-PRIS was SFQ Sado-Masochistic (*r* = .79, *p* < .001).

### Multiple Linear Regression

We conducted a multiple linear regression to gauge which dynamic risk factors were the strongest correlates for M-PRIS Total. As seen in Table 2, all variables in the model were centered, which consisted of the UCLA Loneliness Total, the BPAQ, SFQ Total, SRMA Total, ARS Total, and SRP Total. Results showed a significant model, *F*(6, 108) = 28.6, *p* < .05, which explained 61.4% of the variance. Specifically, BPAQ Total, SFQ Total, and ARS Total were significant (*sr*^2^ = .0543, 0.0499, 0.0488, respectively).

**Table 2:** Multiple Linear Regression of M-PRIS as a Function of Exploratory Variables

Exploratory Variables	*b*	95% CI	*sr*	*p*
		*LL*, *UL*		
Intercept	-27.75			
UCLA Loneliness Total	-0.23	-1.11, 0.65	.061	.60
BPAQ Total	0.87	0.14, 1.60	.054	.02
SFQ Total	0.41	0.21, 0.61	.050	< .001
ARS Total	-0.65	-1.19, 0.12	.049	.02
SRMA Total	0.48	-0.02, 0.98	.083	.06
SRP Total	0.21	-.15, .58	.063	.25

*Note*. *b* = unstandardized regression coefficient; *sr* = semi-partial correlation; *N* = 115, *F*(6, 108) = 28.6, *p* < .001, Adjusted *R*^2^ = .614.

## Discussion

The primary purpose of the study was to investigate potential correlates associated with a proclivity for multiple-perpetrator rape and reconfirm prior findings in the MPR literature. The study was guided by previously mentioned literature, and the primary research question: Do certain correlates have a stronger relationship than others with the proclivity for multiple-perpetrator rape? There were positive significant associations between all exploratory dynamic risk factors and the outcome of interest that ranged from weak to strong. Additionally, multiple linear regression demonstrated three dynamic risk factors to be significant and explained a large variance in a proclivity for MPR. Specifically, BPAQ Total, SFQ Total, and ARS Total were significant in the model and explained a total of 61.4% of the variance in an individual’s proclivity for MPR indicating that they seem to be the most important correlates for MPR proclivity.

### Loneliness

Several studies have found loneliness to be strongly related to lone sexual offending (Hanson & Morton-Bourgon, 2005; Marshall, 2010; Seidman et al., 1994, Wielinga et al., 2021), but no study investigated the relationship between loneliness and MPR. Given the inherently social nature of group offending (Harkins & Dixon, 2010), loneliness and a need for belonging could theoretically be an important influence for willingness to engage in MPR. In our study, loneliness was found to have a weak positive relationship with MPR proclivity, and the sample’s mean was below the midpoint of the scale. The positive correlation provides evidence that, despite loneliness not being a significant correlate of MPR proclivity in the multiple linear regression, an individual’s feelings of loneliness may still have an impact on proclivity for MPR and should be further investigated both by researchers and clinicians. Specifically, understanding feelings of loneliness and how to build meaningful social bonds with others may serve as a protective factor against sexual recidivism. A possible reason for the weak relationship and low mean is that the UCLA Loneliness Scale: SF is not a forensic measure or created to be identified as a risk construct, or the sample has a stronger level of social support and intimacy (Wielinga et al., 2021). Therefore, the feelings of loneliness captured in this assessment may not have been appropriate to identify the true relationship between loneliness and a proclivity for MPR–further strengthening the call for more research into the area.

### Aggressive Behavior Tendencies

Aggressive behavior tendencies have been linked with MPR, with MPRs tending to be more aggressive in nature compared to lone sexual offenders (Hauffe & Porter, 2009; Porter & Alison, 2006; Woodhams & Cooke, 2013). In our study, aggressive behavior tendencies (i.e., physical aggression, verbal aggression, hostility, and anger) were found to have a moderate positive relationship with a proclivity for MPR, which supports prior findings. One potential explanation for the heightened aggressiveness may be rooted in the cognitive appraisal that there is safety and power in numbers, and the victim physically cannot stop the offenders. Through this line of thinking, they may become unbound by any societal or personal restrictions. The findings also suggest that there was no single dimension of aggression that exclusively overpowers another; however, BPAQ Physical Aggression did have the strongest relation among the subscales. To enhance prevention efforts, forensic clinicians should strive to understand how MPR offenders' aggressive behavior tendencies begin and how they cope (or fail to cope) when feelings of hostility or anger arise.

### Deviant Sexual Fantasies

The current study found the strongest overall relationships between deviant sexual interests (i.e., SFQ Total, SFQ Exploratory, SFQ Intimate, SFQ Impersonal, SFQ Sado-Masochistic) and a proclivity for MPR. Additionally, the means were above the midpoints for the total and subscales indicating fairly moderately high levels of these fantasies. Findings further confirm Harkins and Dixon's (2013) Multi-Factorial Theory of Multiple Perpetrator Sexual Offending, which posited that deviant sexual interest is a leading individual factor for MPR. Previous research (e.g., Alleyne et al., 2014) did not include sadomasochism in their studies, which interestingly was found to be the greatest correlate for MPR proclivity out of all the correlates that were tested. MPR can be seen as one of the most violent sexual offending types, which may explain why sadomasochism was found to hold a strong linkage to MPR. A group’s sadomasochism drive may explicitly come out to hurt and feel immense pleasure from the pain they are inflicting.

### Rape-Myth Cognitions

The Multi-Factorial Theory of Multiple Perpetrator Sexual Offending (Harkins & Dixon, 2010) posits the sociocultural factor that rapes myth cognitions, such as justification for sexual violence against women, may influence individuals to potentially participate in MPR. The current study supports the notion as the SRMA Total has a significant correlation with a proclivity in MPR. Alleyne and colleagues (2014) reported similar findings, where rape myth cognitions were a major correlate of MPR proclivity. The lack of significance of the SRMA in the multiple linear regression may be due to collecting data from a community sample. To support this assumption, previous work by Johnson and Beech (2017) found that rapists were significantly more accepting of rape myths than non-sexual offenders and nonoffenders.

### Anger Rumination

No prior research has investigated the relationship between anger rumination and MPR, but Johnson and Beech (2017) discovered higher trait anger in sex offenders than nonoffenders. Anger rumination was selected as a prospective correlate due to its established significance in prior research as a robust predictor of various manifestations of aggressive and violent behaviors (Anestis et al., 2009; Denson, 2013; Vasquez et al., 2012). In the current study, anger rumination (i.e., ARS Total, ARS Angry Afterthoughts, ARS Thoughts of Revenge, ARS Angry Memories, ARS Understanding of Causes) had an overall moderate relationship with proclivity for MPR. As anger rumination was significant in the multiple linear regression, the relationship with MPR proclivity demonstrates the potential need for prevention programs and clinicians to target anger rumination among those who may be at risk for MPR offending. Obtaining a fuller understanding of how anger rumination plays in MPR offending will allow for treatment and prevention programs to better address this correlation with MPR proclivity. Furthermore, results demonstrate that further understanding of how anger rumination, within the context of grievance thinking, is needed to potentially fully understand MPR (Barnett, 2011; Wakeling & Barnett, 2011).

### Psychopathy

Lastly, the four-factor psychopathy model, which consists of Interpersonal Manipulation (i.e., pathological lying, glib, being manipulative), Callous Affect (i.e., lack of remorse and empathy, low affect), Erratic Lifestyle (i.e., impulsivity, recklessness), Antisocial Behavior (i.e., criminal behavior, aggression), was investigated due to its mixed relationship with sexual offending (Darjee, 2019; Hanson & Morton-Bourgon, 2005; Higgs et al., 2019; Moretti et al., 2024; Mouilso & Calhoun, 2013). Psychopathy was not significant in the multiple linear regression, which may be due to the non-clinical and/or forensic sample in comparison to Castellana and colleagues’ (2014) work which found that young offenders scored higher on the PCL-R than non-offenders.

Those interested in MPR offenses seem to reflect multiple psychopathic traits, which supports prior research on sexual offender characteristics. MPR is an extreme form of sexual violence, so individuals with a lack of remorse, high recklessness, and willingness to commit a violent/criminal action do fit a plausible character profile for an MPR offender.

### Limitations and Future Directions

There are a few limitations to the study. First, the study primarily used self-report measures for all variables. The research design was cross-sectional, so causation cannot be determined among any associations. Although self-report measures are efficient, they provoke some concerns. Social desirability bias may have influenced responses (King, 2022), such that loneliness and proclivity for multiple-perpetrator rape are rated in a more positive frame (i.e., answered to perceive self with lower loneliness and proclivity for multiple-perpetrator rape). Furthermore, the M-PRIS measures *interest* in MPR behaviors which may not necessarily translate into *action* should these individuals be placed in a similar situation. Research does indicate that there is a relatively high degree of concordance between fantasy and behavior in relation to paraphilic behavior (Joyal & Carpentier, 2022; Seto et al., 2021) and sexual aggression (Birke & Bondü, 2023), this is an untested assumption for MPR proclivity. Therefore, future research on concordance and discordance between proclivities and actual behaviors may be important future directions and should be considered in future studies. The sample size was also relatively small, meaning that some statistical tests may be underpowered and the true effect is not present (Button et al., 2013). Therefore, study results should be considered carefully and future research should strive to collect a larger sample.

Although the study presented interesting findings, there are ways to improve in future studies. To further examine the associations between the dynamic risk factors and proclivity for multiple-perpetrator rape, future studies should employ more sophisticated designs and statistical procedures in order to establish potential causal relationships. Future studies could also interview those charged or incarcerated for MPR offenses to establish their experiences of how these factors may have played a role in their behaviors. Lastly, future studies should obtain a larger sample that would yield more power and a more diverse sample that includes more racial and ethnic individuals.

### Implications

One implication is for clinicians to target various types of aggression, deviant sexual fantasies, and anger rumination in therapy among those with an interest to commit MPR to potentially reduce the urge to commit the action. Educational and preventive initiatives aimed at addressing sexual violence behaviors may also gain insights into individuals prone to engaging in such behaviors, where these programs seek to diminish the likelihood of MPR occurrences. The results have important implications for clinical, education, and prevention efforts related to sexual violence (e.g., Bringing in the Bystander; Banyard et al., 2004).

### Conclusion

Multiple-perpetrator rape is an underreported form of sexual violence (Chambers et al., 2010), and understanding the potential dynamic risk factors of individuals may be a solution to reduce the prevalence. The findings from this study demonstrate that multiple dynamic risk factors correlate to a proclivity for MPR. The results contribute to the sexual violence literature by identifying potential dynamic risk factors (i.e., *loneliness, aggressive behavior tendencies, anger rumination, psychopathy, deviant sexual interests, and rape myths acceptance*) as major correlates for a proclivity for MPR and reconfirming prior research findings on MPR.

## Data Availability

The data/coding that supports the findings of this study is available from the corresponding author.

## Appendix A

**Table A1:** M-PRIS Scale and Subscale Means

Scale	*M*	*SD*	Cronbach’s α
Initiation	2.72	1.47	.91
Sexual Arousal	2.66	1.64	.78
Behavioral propensity	2.76	1.55	.77
Enjoyment	2.65	1.60	.75
Intimidation	2.77	1.55	.92
Sexual Arousal	2.73	1.71	.82
Behavioral propensity	2.72	1.56	.73
Enjoyment	2.80	1.70	.80
Overall	2.75	1.48	.96

*Note.* *Paired samples *t*-test measuring Initiation vs. Intimation scales were found to be non significant (*t*(110) = -1.06, *p* = .293).

## Appendix B

**Table B1:** M-PRIS Distribution of Responses for Each Vignette

M-PRIS Vignette Number and Type	Definitely Negative						Definitely Positive
	1 (%)	2 (%)	3 (%)	4 (%)	5 (%)	6 (%)	7 (%)
Vignette 1: Initiation							
Sexual Arousal	43	15	11	10	5	8	8
Behavior propensity	37	15	9	17	7	7	8
Enjoyment	45	11	14	15	6	4	6
Vignette 2: Intimidation							
Sexual arousal	40	12	10	15	5	10	8
Behavior propensity	44	11	16	13	3	5	8
Enjoyment	41	14	11	14	4	6	11
Vignette 3: Initiation							
Sexual arousal	43	17	14	11	1	6	8
Behavior propensity	40	18	15	11	8	4	5
Enjoyment	47	16	11	10	4	4	9
Vignette 4: Intimidation							
Sexual arousal	50	10	11	10	4	6	8
Behavior propensity	46	11	16	13	4	5	5
Enjoyment	44	15	10	8	6	9	8
Vignette 5: Initiation							
Sexual arousal	45	8	20	10	3	6	8
Behavior propensity	42	10	20	12	7	3	6
Enjoyment	47	10	10	13	6	7	7
Vignette 6: Intimidation							
Sexual arousal	45	10	17	12	2	9	6
Behavior propensity	41	11	11	16	9	3	10
Enjoyment	41	12	17	13	4	8	5
